# Adding dendritic cell-immunotherapy for post-transplant hepatocellular carcinoma recurrence

**DOI:** 10.3389/fimmu.2025.1589634

**Published:** 2025-08-04

**Authors:** Wei-Chen Lee, Chih-Hsien Cheng, Tsung-Han Wu, Yu-Chao Wang, Jin-Chiao Lee, Hao-Chien Hung, Chen-Fang Lee, Ting-Jung Wu, Hong-Shiue Chou, Kun-Ming Chan

**Affiliations:** ^1^ Division of Liver and Transplantation Surgery, Department of General Surgery, Chang-Gung Memorial Hospital, Linkou, Taiwan; ^2^ Chang-Gung University College of Medicine, Taoyuan, Taiwan

**Keywords:** DC, dendritic cell, hepatocellular carcinoma, liver transplantation, tyrosine kinase inhibitor, immune checkpoint inhibitor

## Abstract

**Background:**

Hepatocellular carcinoma (HCC) recurrence after liver transplantation is frequently multiple and extrahepatic, and with a poor prognosis. The therapeutic effects of current medications for post-transplant HCC recurrence are limited. This study assessed whether outcomes could be improved by adding dendritic cell (DC)immunotherapy to the treatment regimen.

**Methods:**

Eleven patients treated with tyrosine kinase inhibitors and DC-immunotherapy for post-transplant HCC recurrence between 2020 and 2024 were included. DC were propagated from peripheral blood monocytes and pulsed with tumor lysate. Historical data of patients (n =23) with tyrosine kinase inhibitors for post-transplant HCC recurrence between 2009 and 2020 were collected as a reference.

**Results:**

Seven male and four female patients were included in this study. The median (interquartile) tumor recurrence time after transplantation was 35.0 (7.4-55.3) months. The median number of DC-immunotherapy were 5 ranged from 3 to 10, and the median number of cells admitted was 29.5x10^6^ cells ranged from 16.0 to 137.2 x10^6^ cells. Responses to DC-immunotherapy included nine stable diseases and two progressive diseases. No adverse effects related to DC treatment were observed. The 1-, 2- and 3- year survival rates were 70.7%, 40.4%, and 40.4%, respectively, compared to 52.5%, 17.4%, and 8.7%, respectively, for patients treated with tyrosine kinase inhibitors only (*p* = 0.050).

**Conclusion:**

DC immunotherapy is a safe treatment for transplant recipients with HCC recurrence. Adding DC-immunotherapy to the treatment regimen could prolong the survival of some patients.

## Introduction

Hepatocellular carcinoma (HCC) had been a relative contraindication for liver transplantation because HCC was easy to recur after liver transplantation. Until the so-called Milan criteria of liver transplantation were established for HCC, liver transplantation was resumed for HCC treatment (1). Currently, liver transplantation is the treatment of choice for HCC and yields the best outcome among the therapeutic modalities if the tumors are within the Milan criteria (2). Nevertheless, the Milan criteria are frequently challenged to restrict HCC patients from undergoing liver transplantation. Hence, the criteria for liver transplantation for HCC are extended to the University of California, San Francisco (UCSF) criteria, up-to-7 criteria, and various other criteria (3–5). However, the extension of criteria will expense the outcomes of liver transplantation for HCC, as tumor recurrence will increase (6, 7).

HCC recurrence after liver transplantation is a critical issue because treatment is troublesome and the prognosis is very poor. In the literature, the tumor recurrence rate after liver transplantation was 13-17% (8, 9). When these criteria are extended, the tumor recurrence rate is expected to increase. Post-transplant recurrent HCC is usually extra-liver metastasis, and effective treatments for extra-liver metastasis are still lacking. Sorafenib, a tyrosine kinase inhibitor (TKI), was introduced in 2008 to treat advanced HCC. Patient survival can be significantly prolonged, although the objective response rate is low (10, 11). Nivolumab, an immune checkpoint inhibitor (ICI), was used to treat advanced HCC with a 15-20% objective response (12). Recently, a combination of atezolizumab and bevacizumab achieved a 30% of objective response rate for advanced HCC and could be used to treat extra-liver metastases (13). Nevertheless, ICI is concerned to be used in organ transplant patients because ICI may induce acute rejection and result in graft loss (14–16). Hence, TKI remains the main therapeutic option for post-transplant HCC recurrence (17, 18).

Dendritic cells (DC) are the most potent antigen-presenting cells and can process tumor-specific or associated antigens to activate cytotoxic T-cells and undergo anti-tumor immunity. Theoretically, cancer cells develop due to transformation of cells as a consequent of enhanced oncogenes or loss of tumor suppressor genes. Cancer cells may express tumor-specific or tumor associated antigens due to genetic change. DC can pick up these antigens to stimulate antigen-specific cytotoxic T-cells and attack cancer cells. DC has been applied to treat various cancer with promising effects and without major adverse effects (19). For early stage HCC, DC can be used as an adjuvant therapy after liver resection to increase disease-free and overall survival (20, 21). For advanced HCC, DC-therapy could increase count of T-lymphocyte and improve overall survival (22). In our previous study, DC vaccination could achieve a 12.9% of objective response rate and a 67.7% of disease control rate (23). In a meta-analysis, DC-based immunotherapy for HCC could increase 1.9 folds of progression-free survival and 1.72 folds of overall survival compared to the control (24). Post-transplant recurrent HCC is usually advanced and metastatic, and DC may be usable to treat these tumors.

When HCC recurred after liver transplantation, the median survival was only 7–16 months (8, 9, 25). For improving survival, some effective treatments must be added. Since DC could be used to treat advanced HCC, we added tumor lysate-pulsed DC to supplement TKI for post-transplant HCC recurrence in this study. Herein, we examined the preliminary therapeutic results of TKI supplemented with DC for post-transplant HCC recurrence.

## Patients and methods

### Patients

Patients who had post-transplant HCC recurrence and were treated with TKI supplemented with DC therapy from 2020 to 2024 were included in the study. Patient profiles, tumor characteristics, and post-recurrence treatments were collected for analysis. The objective of this study was to assess whether DC-immunotherapy was safe and could prolong patient survival when HCC recurred after liver transplantation. DC-immunotherapy was approved by the Ministry of Health and Welfare, Taiwan, under special regulations. This study conformed to the ethical guidelines of the 2000 Declaration of Helsinki and was approved by the institutional review board of Chang-Gung Memorial Hospital, Linkou Main Branch. (IRB No.202300279BO).

### Clinical diagnosis and criteria of liver transplantation

The diagnosis of HCC was dependent on tumor lesions on dynamic computed tomography (CT) or magnetic resonance imaging (MRI) with a typical vascular pattern, while AFP was ≥ 200ng/ml, or tumor lesions with a typical vascular pattern in two imaging studies. A typical vascular pattern was defined as contrast uptake during the arterial phase and washout during the venous and late phases. The criteria for HCC for transplantation were Milan criteria (1) for deceased liver and UCSF criteria (3) for living donor liver transplantation.

### Post-transplant immunosuppressive regimen

After liver transplantation, immunosuppression was induced by an immunosuppressive regimen consisting of steroids, tacrolimus, mycophenolate mofetil, and everolimus. Methylprednisolone was not administered during the operation. Postoperatively, methylprednisolone was tapered from 200 mg/day to 40 mg/day over five days and discontinued within three months after the operation. Tacrolimus was administered orally on post-transplant day 2 or 3, when renal function was restored. Therapeutic trough levels of tacrolimus were targeted at 5–8 ng/ml which was achieved within 7 days after transplantation. Tough levels of tacrolimus were kept at 3-5ng/ml in the long-term. Mycophenolate mofetil (MMF; 0.5–1 g/day) or everolimus (1 mg/day) was given orally one month after transplantation.

### Clinical follow up

Liver tumor recurrence was screened for every liver transplant patient whose explant liver showed HCC. A-fetoprotein (AFP) levels were measured every 3 months after transplantation. Liver ultrasonography was performed every three months in the first three post-transplant years and then every six months. The screen activities lasted forever. CT was performed every 3 months in the first year and every 6 months in the 2^nd^ year. However, CT can be performed anytime if needed. Tumor recurrence was defined as a tumor in the liver with a typical vascular pattern or extra-liver metastases.

### Generation of DC and DC administration

The propagation of DC proceeded as described in our previous report (23). Briefly, peripheral blood, 50–60 ml, was obtained for DC propagation. Monocytes were isolated and suspended in serum-free AIM-V medium (Life Technologies, Graitherburg, MD, USA) supplemented with recombinant granulocyte-macrophage colony-stimulating factor (1000u/ml; R&D System Inc., Minneapolis, MN, USA) and interleukin (IL)-4 (1000u/ml; R&D System Inc., Minneapolis, MN, USA). Two days before harvesting DC, the DCs were pulsed with autologous tumor lysates and matured with cocktail cytokines. Before DC administration, DC must be negative for bacteria, mycoplasma, and high expression (> 60%) of CD40, CD80, CD83, CD86, and HLA-DR. Fresh 50–60 ml of peripheral blood was drawn to isolate monocytes for each time of DC propagation. The peripheral blood monocytes were not stored. The DCs yielded in each preparation was administrated within 4 hours, and were not stored, either. The number of cells infusion depended on the cell number yielded in each DC preparation. Three courses of DC were administered intravenously at 2-week interval. DC was boosted if the patient was willing to continue DC therapy.

### Tumor lysate preparation

Tumor specimens were obtained via biopsy or local surgical excision. Tumor cells were dispersed into a single-cell suspension (2x10^6^ cells/ml). These tumor cells were lysed using three cycles of snap freeze-thawing to obtain the tumor lysate. The large particles were removed by centrifuge (600rpm for 5 min). The lysate was preserved in –20°C freezer until it served as a tumor antigen.

### Immune cell and tumor response to DC-immunotherapy

To examine immune cell response to DC-therapy, the percentage of CD4^+^ and CD8^+^ cells in peripheral lymphocytes were measured prior to and one month after DC-therapy by flow cytometry after stained by florescence-conjugated monoclonal antibodies. The tumor response to DC-immunotherapy was assessed using the Response Evaluation Criteria in Solid Tumors 1.1 (RECIST 1.1) and modified RECIST (mRECIST) (26). Briefly, complete response (CR) was defined as disappearance of all target visible lesions, partial response (PR) was defined as at least a 30% decrease from the baseline sum of the longest diameter, stable disease (SD) was defined as no significant change in the size of target lesions, and progressive disease (PD) was defined as ≥20% increase in the sum of the longest diameter or appearance of any new lesions.

### Biostatistics

Categorical variables were compared using the Chi-square or Fisher’s exact tests. The significance of differences between different groups was determined using paired or unpaired Student’s t-tests. Estimated survival rates were calculated using the Kaplan-Meier method. All statistical analyses were performed using SigmaPlot 14 for Windows (Systat Software, Inc., San Jose, CA, USA). *P* < 0.05 was considered statistically significant.

## Results

### Patients

Eleven patients (seven males and four females) with TKI and DC-immunotherapy for post-transplant HCC recurrence were included. The median (interquartile) age at tumor recurrence was 56.4 (54.9-64.1) years ranging from 50 to 72 years. The median (interquartile) tumor recurrence time after transplantation was 35 (7.4 -55.3) months ranging from 3 to 114 months. Among them, only one patient had recurrent HCC limited to the liver, and all other patients had extra-liver metastases (**Table 1**). Moreover, most of the recurrent tumors were multiple; therefore, lung metastasis was observed in 7 (63.6%) patients, intrahepatic metastasis in 5 (45.5%) patients, lymph node metastasis in 3 (27.3%) patients, adrenal gland metastasis in 2 patients, bone metastasis in 1 patient, abdominal muscle metastasis in 1 patient, and inferior vena cava thrombus in 1 patient.

**Table 1:** The characteristics of the 11 patients with DC-immunotherapy.

Patients	gender	Age	Op	Time to recurrence (m)	Site of tumor recurrence	TKI	Response to DC	Survival time (m)	Outcomes
1	F	57	LDLT	35	Mediastinal lymph nodes, lung, liver, kidney	Sora →Reg	PD	42.5	D
2	M	57	DDLT	114	lung, liver	Sora	SD (minor reaction)	52.5	A
3	M	50	DDLT	9	Liver, periportal lymph nodes	Sora → Reg	SD	14	D
4	M	59	LDLT	24	Lung, rectal abdominal muscle	Len	SD (minor response)	18	D
5	M	64	LDLT	48	lung	Len	SD	12	D
6	F	58	LDLT	22	liver	Len	SD	57	A
7	F	72	LDLT	57	Lung, left adrenal gland	Len	SD	24	D
8	M	61	DDLT	35	Iliac bone	Sora	SD	51	A
9	M	53	LDLT	3	Lung	Len	SD	11	D
10	F	56	LDLT	4.5	Lung Retroperitoneal lymph nodes	Len	PD	4.5	D
11	M	66	LDLT	90	Liver, right adrenal gland, IVC thrombus	Reg	SD	6	A

IVC, inferior vena vaca; Op, operation; DDLT, deceased donor liver transplantation; LDLT, living donor liver transplantation; TKI, tyrosine kinase inhibitor; Sora, sorafenib; Reg, regorafenib; Len, lenvatinid; PR, partial response; SD, stable disease; PD, progressive disease; N/A, not assess; D, dead; A, alive.

### Phenotype of DC and DC administration

**Figure 1:**
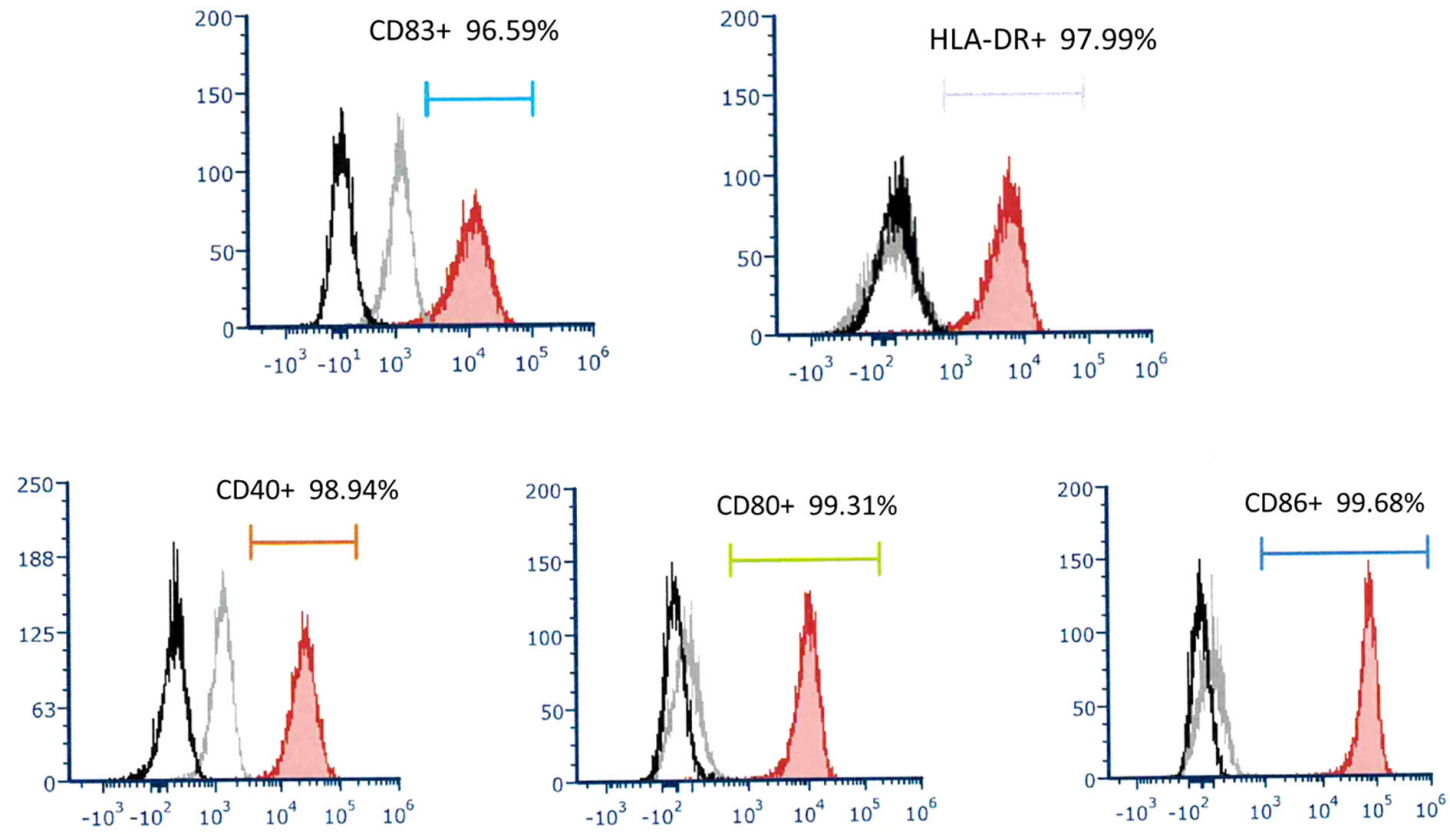
A representative of surface molecular expression and MHC class II antigen on DC. The DC applied to treat recurrent HCC expressed high levels of CD83, HLA-DR, CD40, CD80, and CD86.

To determine the phenotype of DC in this culture system, they were stained with fluorescence-conjugated monoclonal antibodies and analyzed by flow cytometry. DCs express high levels of CD40, CD80, CD86, and HLA-DR. This DC was mature and expressed high levels of CD83. (**Figure 1**) Under our DC propagation methods, the median DC (interquartile) number yielded in each preparation was 3.11 (2.51-5.01) x10^6^ cells ranging from 1.60 to 13.72 x10^6^ cells.

### Outcomes of treatments

**Figure 2:**
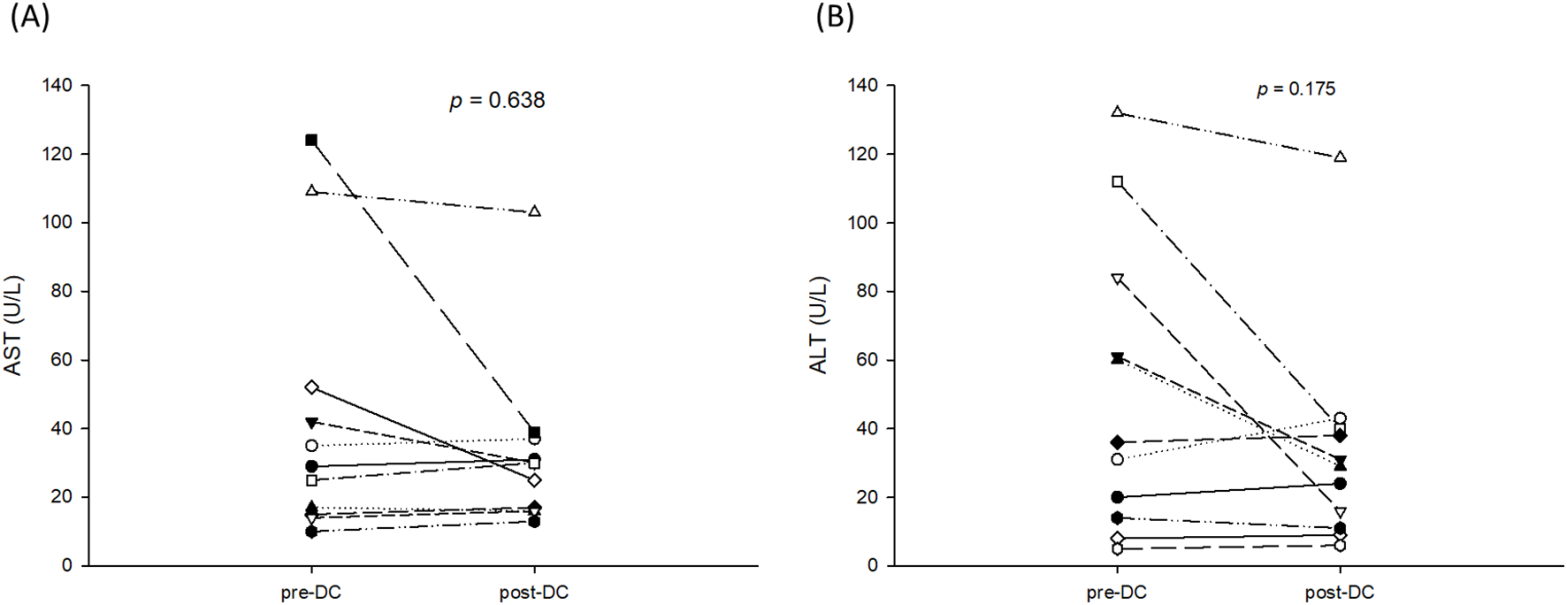
AST and ALT levels prior to and after 3 courses of DC-immunotherapy. **(A)** The median (interquartile) aspartate aminotransferase (AST) level prior to DC therapy was 29 (15-52)U/L and 30 (16-37)U/L after DC therapy (p = 0.638). **(B)** The median (interquartile) alanine aminotransferase (ALT) level was 36 (14-84)U/L prior to DC therapy and 29 (11-40)U/L after DC therapy (p = 0.175).

**Figure 3:**
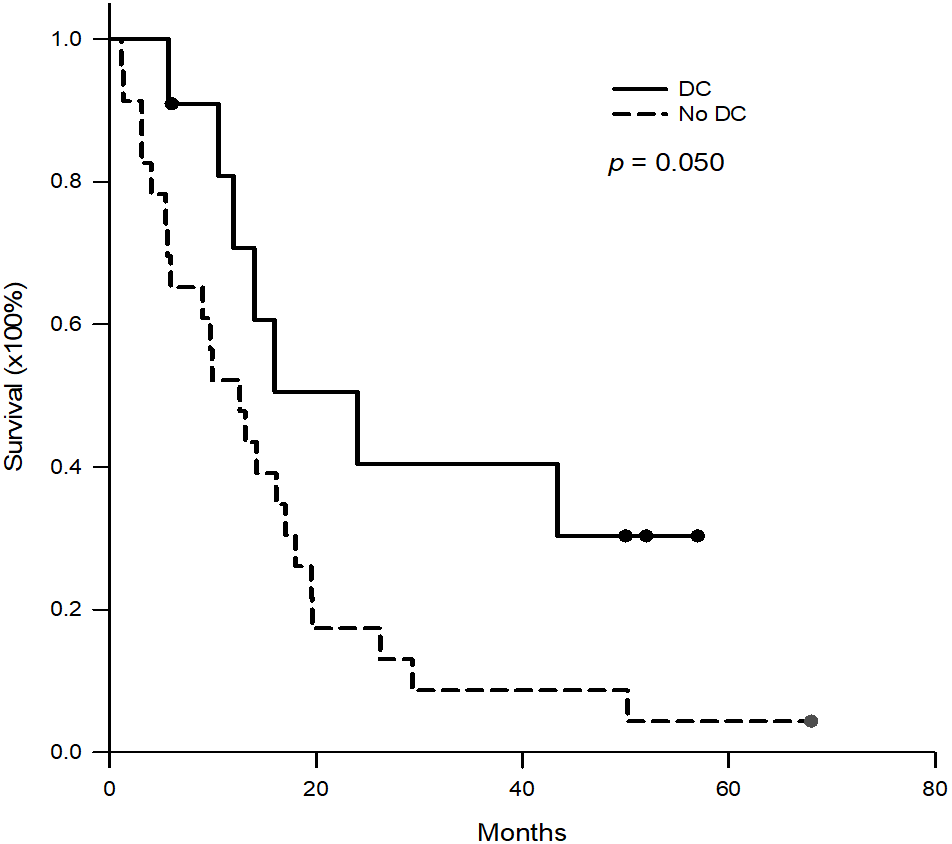
Kaplan-Meier survival curves of the patients with DC-immunotherapy and historical patients without DC-immunotherapy. For the patients with DC-immunotherapy, the 1-, 2-, and 3- year survival rates were 70.7%, 40.4% and 40.4%, respectively. For historical patients without DC-immunotherapy, the 1-, 2-, and 3- year survival rates were 52.2%, 17.4% and 8.7%, respectively. (p = 0.050).

DC-immunotherapy was introduced to treat post-transplant HCC recurrence at this institute in August 2020. All 11 patients with post-transplant HCC recurrence underwent regular treatment with TKI at first, then followed by DC-immunotherapy. The median (interquartile) time from tumor recurrence to DC-immunotherapy was 2.5 (2–9) months ranging from 1 to 24 months. The median course of DC was 5, with a range from 3 to 10. The total number of cells admitted was 31.1x10^6^ cells ranged from 16.0 to 137.2 x10^6^ cells. To assess alteration of T-cells prior to and after DC-immunotherapy, the percentage of CD4^+^ and CB8^+^ cells in peripheral blood lymphocytes were measured. The median (interquartile) percentage of CD4^+^ in T-lymphocytes was 32.2 (29.1- 40.1)% prior to DC therapy, and 32.1 (25.94 -38.2)% one month after DC-therapy (p = 0.625). The median (interquartile) percentage of CD8^+^ in T-lymphocytes was 44.3 (36.4- 51.7)% prior to DC therapy, and 45.3 (41.0 -58.2)% one month after DC-therapy (p = 0.137). Among 11 patients, only 5 patients had elevated levels of AFP. The median (interquartile) level AFP was 39 (19.2 -1125.4) ng/ml prior to DC-therapy, and 71.8 (30.6 – 180.5) ng/ml after DC-therapy (p = 0.137). To assess the responses to DC-immunotherapy, nine patients had stable disease and two had progressive disease. No adverse effects related to DC treatment were observed. The median (interquartile) aspartate aminotransferase (AST) levels prior to DC therapy were 29 (15-52)U/L and 30 (16-37)U/L after DC therapy (**Figure 2A**, p = 0.638). The median (interquartile) alanine aminotransferase (ALT) levels prior to DC therapy were 36 (14-84)U/L and 29 (11-40)U/L after DC therapy (**Figure 2B**, p = 0.175). The concomitant treatments for these patients included pembrolizumab in three patients (two prior to and one after DC-immunotherapy), surgical excision in two patients, radiotherapy in four patients, and transcatheter arterial chemoembolization (TACE) in three patients. For the three patients treated with pembrolizumab, the pembrolizumab courses were 3, 4, and 6, respectively. None of the patients had a tumor response to treatment, and one patient had acute rejection with elevation of liver function. The estimated median (interquartile) survival for these 11 patients was 24 (12-not reach) months. The 1-, 2-, and 3- year survival rates were 70.7, 40.4, and 40.4%, respectively. (**Figure 3**)

### Neutrophil to lymphocyte ratio

**Figure 4:**
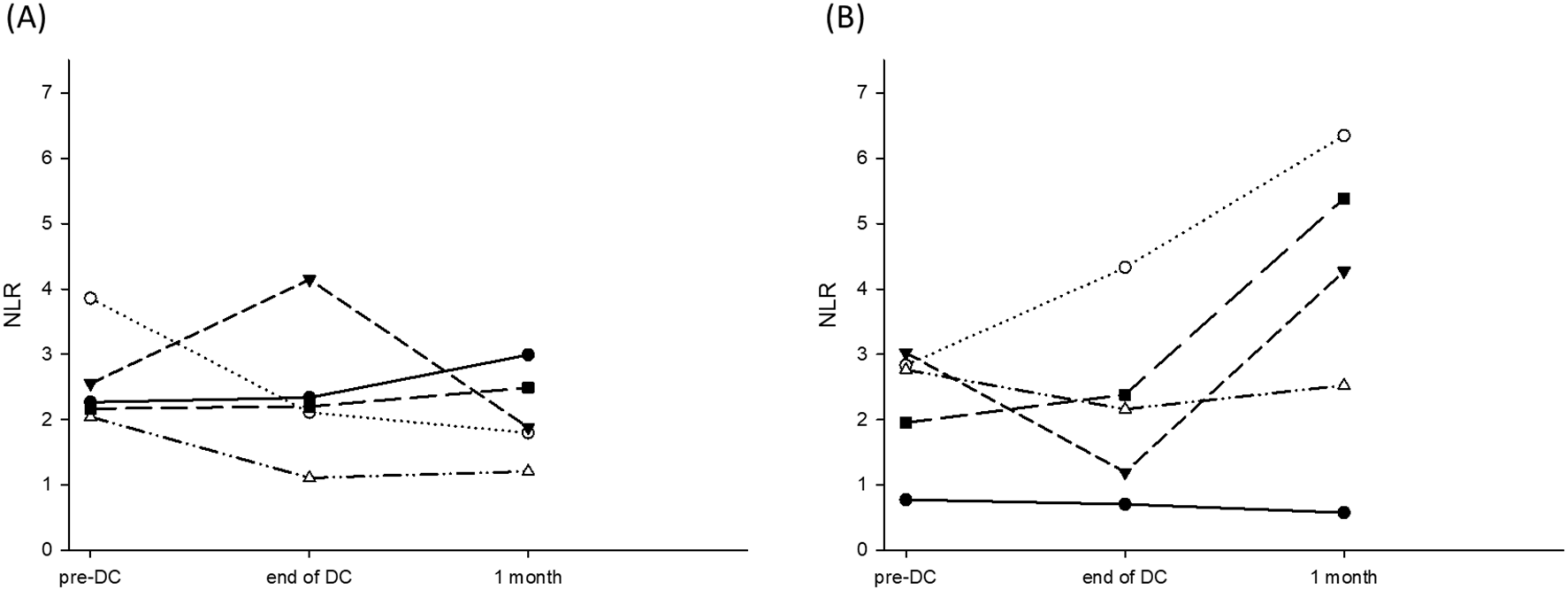
NLR prior to DC-therapy, at the end of DC-therapy and one month after DC-therapy. **(A)** NLR for the patients survived ≥24 months was 2.58 ± 0.74, 2.18 ± 0.73, and 1.93 ± 0.50 prior to DC-therapy, at the end of DC-therapy and one month after DC-therapy, respectively (p = 0.281). **(B)** NLR for the patients survived <24 months was 2.27 ± 0.93, 2.15 ± 1.40, and 3.822 ± 2.31 prior to DC-therapy, at the end of DC-therapy and one month after DC-therapy, respectively (p = 0.522).

To determine whether NLR could be used as a reference to predict the efficacy of DC therapy and outcomes, NLR was measured prior to DC therapy, at the end of DC-therapy and one month after DC therapy. Patients were divided into those who survived for ≥24 months and those who survived for <24 months. The NLR for the patients who survived ≥24 months was 2.58 ± 0.74, 2.18 ± 0.73, and 1.93 ± 0.50 prior to DC therapy, at the end of DC-therapy and one month after DC-therapy, respectively (**Figure 4A**, p = 0.281). The NLR for patients who survived <24 months was 2.27 ± 0.93, 2.15 ± 1.40, and 3.822 ± 2.31, respectively, prior to DC therapy, at the end of DC-therapy, and one month after DC therapy, respectively (**Figure 4B**, p = 0.522). Although the difference in NLR before and after DC therapy did not reach statistical significance in both groups of patients due to the small number of patients, NLR seemed to increase in patients with survival <24 months at one month after DC therapy.

### Historical patients for comparison

The patients with post-transplant HCC recurrence between 2009 and 2020 were collected and reviewed (**Table 2**). The median (interquartile) age at tumor recurrence was 57.3 (49.7-64.3) years. The median (interquartile) tumor recurrence time after transplantation was 13.8 (9.8-27.6) months. Most of the recurrent tumors were multiple and extra-hepatic, such as lung metastasis in 14 (60.9%) patients, intra-liver metastasis in 6 (26.1%), lymph node metastasis in 6 (26.1%), bone metastasis in 6 (26.1%), intra-abdominal carcinomatosis in 5 (21.7%), inferior vena cava/right atrium tumor thrombus in 3 (13.0%), and adrenal gland metastasis in 2 (8.7%). 22 patients received sorafenib, and one received regorafenib. For the 23 patients, the median (interquartile) survival was 12.6 (5.4-19.5) months. The 1-, 2-, and 3- year survival rates were 52.2%, 17.4%, and 8.7%, respectively, which were lower than those of patients receiving DC therapy (**Figure 3**, p = 0.050).

**Table 2:** The characteristic of the patients with DC or without DC-therapy.

Variables	DC (n = 11)	No DC (n = 23)	p
Sex (M/F)	7/4	21/2	0.07
Age (years)	56.4 (54.9-64.1)	56 (50-64)	0.386
Median (IQR)			
Transplant type			0.782
deceased whole liver	2	4	
deceased split liver	0	1	
living donor	9	18	
Viral hepatitis			0.328
B (+)	10	16	
C (+)	0	3	
B (-) C (-)	1	4	
Tumor			
Milan (in/out)	4/7	8/15	1.000
UCSF (in/out)	6/5	13/10	1.000
Vascular invasion (+)	8 (72.7%)	10 (43.5%)	0.146
AFP ≥ 400ng/ml	0	2 (8.7%)	1.000
AFP ≥ 1000ng/ml	0	1 (4.3%)	1.000
Grade			
I	2	3	0.287
II	4	11	
III	2	6	
IV	3	3	
Time to recurrence (months), median (IQR)	35 (7.4-55.3)	13.8 (9.8-27.6)	0.122
TKI			<0.001
Sorafenib	3	22	
Regorafenib	2	1	
lenvatinib	6	0	
Concurrent treatment			
TACE	1 (12.5%)	2 (8.7%)	
local excision	2 (25.0%)	10 (43.5%)	
radiotherapy	2 (25.0%)	2 (8.7%)	

UCSF, University of California San Francisco; AFP, α-fetoprotein; IQR, interquartile range; TACE, transcatheter arterial chemoembolization.

### Case presentation

**Figure 5:**
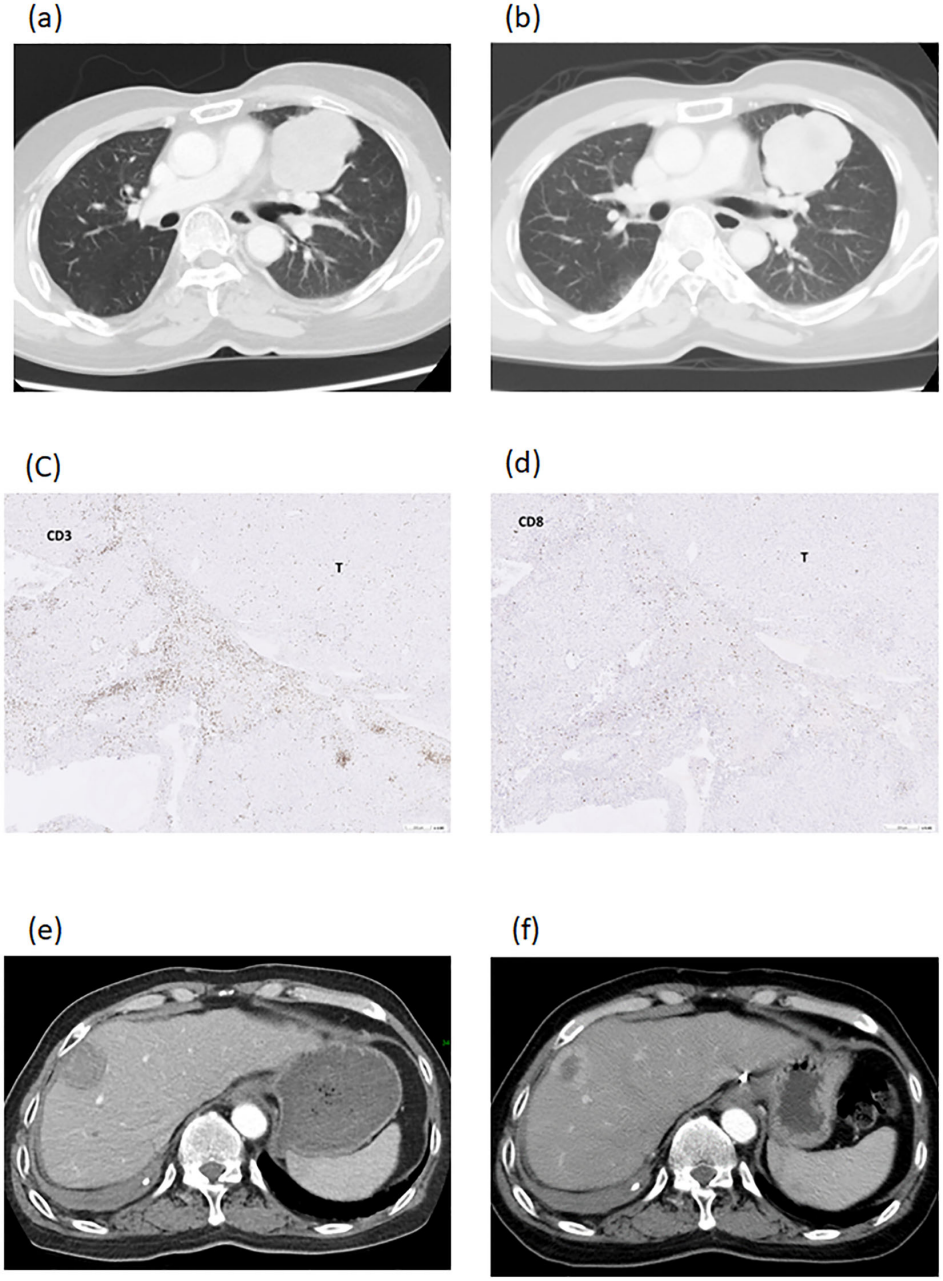
Imaging studies of patient number 2. A recurrent and metastatic tumor, 64.0mm in diameter, in the lung and attached to pericardium **(a)**. The lung tumor diameter was decreased to 59.5mm and freed from the pericardium after DC-therapy **(b)**. The lung tumor was excised and showed necrotic with CD3^+^ cells **(c)** and CD8^+^ cells **(d)** at tumor border. The recurrent tumor in the liver was 33.3mm in diameter **(e)**. The diameter of liver tumor was decreased to 26.3mm after DC-therapy **(f)**.

Case 2 was a 57-year old male patient who had a deceased liver transplantation for hepatitis B cirrhosis and HCC at his 40s. Recurrent and metastatic tumors were seen in the lung (solitary and attached to pericardium, 64.0mm in diameter, **Figure 5a**) and the liver (solitary, 33.3mm in diameter, **Figure 5e**). Lung tumor biopsy was done to serve as tumor antigens to proceed DC-therapy. The lung tumor diameter was decreased from 64.0mm to 59.5mm and freed from the pericardium after 3 courses of DC (**Figure 5b**). Then, the lung tumor was excised and served as tumor antigens to proceed 3 additional coursed of DC-immunotherapy. The pathological figures of lung tumor showed tumor necrosis with CD3^+^ and CD8^+^ cells at tumor border (**Figures 5c, d**). After 3 additional DC-therapy, the diameter of liver tumor was decreased from 33.3mm to 26.3mm (**Figures 5e, f**). Later on, liver resection was performed to excise the tumor. Now, the patient is freed from HCC for 3 years.

**Figure 6:**
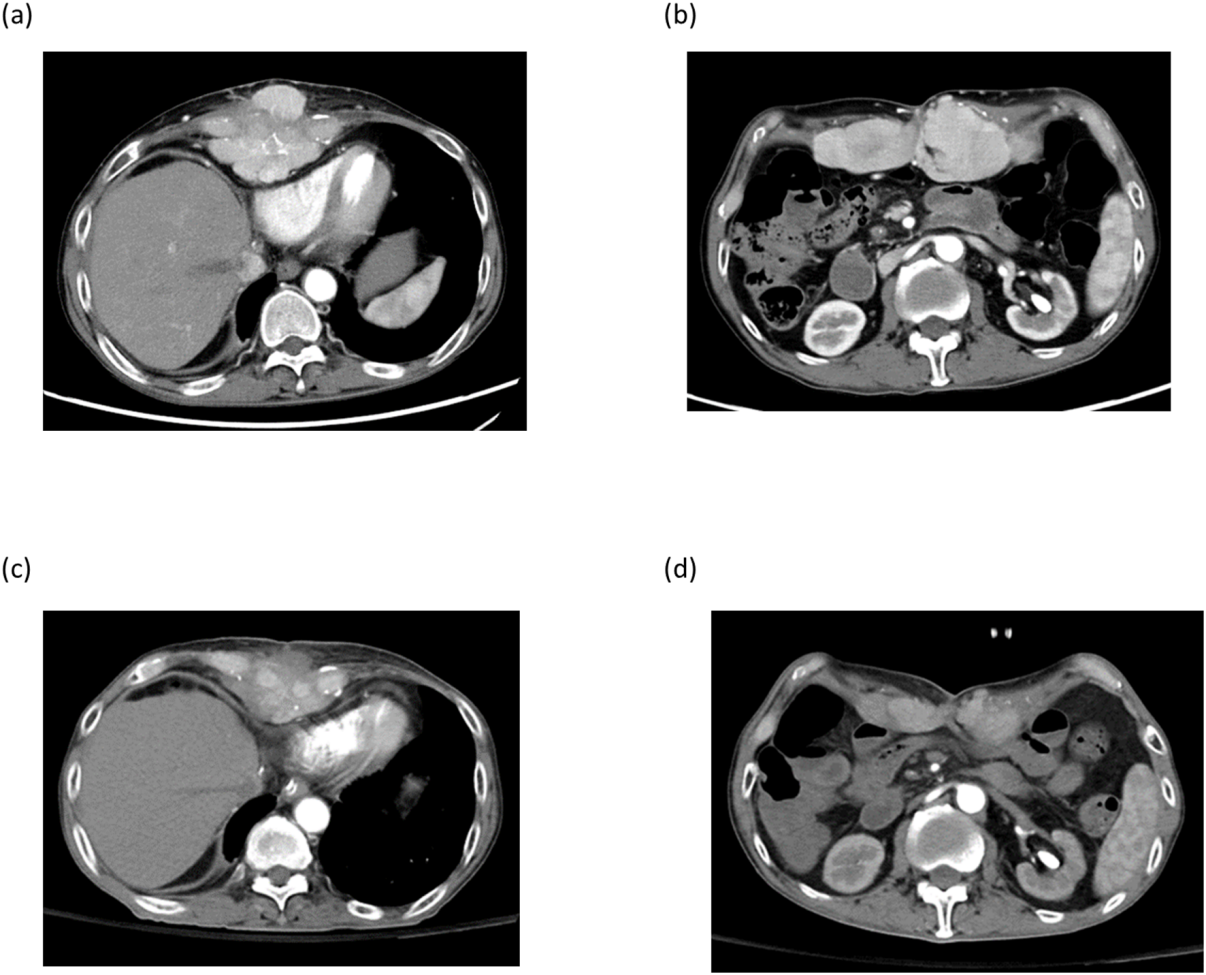
Imaging studies of patient number 4. Post-transplant HCC recurrence at xyphoid process **(a)** and abdominal rectal muscle **(b)**. After 9 courses of DC-immunotherapy, tumors at xiphoid process and abdominal rectal muscle regressed **(c , d)**.

Case 4 was a 59-year-old male with hepatitis B who had tumor recurrence in the lung and abdominal rectal muscle 24 months after living donor liver transplantation. A combination of pembrolizumab and lenvatinib has been used to treat tumor recurrence. After 3 courses of pembrolizumab, acute rejection occurred with elevation of AST and ALT; hence, the treatment was discontinued. Imaging studies showed tumor progression, and the sum of target lesion diameters increased from 152.0 mm to 189.62 mm. Instead of pembrolizumab, the treatment was replaced with DC immunotherapy. After 9 courses of DC infusion, the disease was stabilized and the sum of the target lesion diameters decreased to 140.8 mm. (**Figure 6**) Unfortunately, the patient got Covid-19 and died of respiratory failure 18 months after the HCC recurrence.

## Discussion

Liver transplantation is indicated for HCC to yield the best outcomes among HCC treatment modalities. However, post-transplant HCC recurrence is a critical issue because most recurrent tumors are extrahepatic and multiple, and effective treatments are lacking. The prognosis of post-transplant HCC recurrence is very poor, with a median survival of only 7–16 months (8, 9, 25). Hence, the indication of liver transplantation is limited to the Milan criteria or only allows modest extension of the criteria in order to minimize tumor recurrence (1, 3, 4, 6). In our liver transplantation program, the criteria for liver transplantation are limited to the Milan criteria for deceased liver transplantation or the UCSF criteria for living donor liver transplantation. However, there is a discrepancy between clinical imaging and pathological findings. In this study, 63.6% of the patients were beyond the Milan criteria and 45.5% of the patients were beyond the UCSF criteria based on pathological findings. Of these patients, 72.5% had microvascular invasion, which was difficult to identify before transplantation. Post-transplantation screening for tumor recurrence is essential for patients with HCC (9, 27); in particular, tumors had vascular invasion or were beyond the criteria.

Post-transplant HCC recurrence is frequently extrahepatic and multiple in nature. Treatment of post-transplant HCC recurrence is difficult. Before TKI was approved for the treatment of advanced HCC, treatments for post-transplant recurrent HCC were lacking. Sorafenib was the first TKI approved for the treatment of advanced HCC, and was introduced in Taiwan in 2008. Since then, sorafenib has been the first-line treatment for patients with posttransplant HCC recurrence. Lenvatinib is another TKI approved for the treatment of advanced HCC, as the treatment results were not inferior to sorafenib in a clinical trial. Although the overall survival of patients with advanced HCC was not different between sorafenib and lenvatinib treatment, lenvatinib had a higher objective response rate and longer progression-free survival than sorafenib (28). Hence, the treatment for post-transplant recurrent HCC has shifted from sorefenib to lenvatinib at our institute. In 2019, the Ministry of Health and Welfare in Taiwan opened cell therapy under special regulations. Therefore, DC immunotherapy was added to joint TKI for post-transplant HCC recurrence.

DCs are the most potent antigen-presenting cells and can process tumor-specific or associated antigens to activate antigen-specific cytotoxic T-cells. The development of cancer cells is recognized as cell transformation due to a consequence of enhancement of oncogenes or loss of tumor suppressor genes. DC has been applied to treat various cancer with promising effects and without major adverse effects. DC has been used as a cancer vaccine to decrease HCC recurrence and prolong patient survival after liver resection, reported by Sun TY et al. (20) DC combined with cytokine-induced killer cells also has been used to decrease post-hepatectomy tumor recurrence (21), or tumor response/patient survival when combined with trans-arterial chemoembolization (29). Besides usage of DC-therapy in early and intermediate stage HCC, DC was used to treat advanced HCC. In a meta-analysis study, DC could increase progression-free and overall survival, compared to traditional treatments (24). As the therapeutic effects of TKI for post-transplant recurrent HCC were limited, DC may be added to the treatment regimen to increase therapeutic efficacy and improve patient survival.

DC immunotherapy improves the survival of patients with post-transplant HCC recurrence. When DC-immunotherapy was added into the treatment regimen, the median survival was increased from 12.6 month in historical patients to 24.0 months. The 1-, 2-, and 3-year survival rates also increased. The median survival for our historical patients was similar to that reported by Rajendran et al., who reported that the survival of patients with post-transplant HCC recurrence was 10–13 month (9). Dr. Mahmud et al. also reported that the median survival after HCC recurrence was 13.2 months (25). TKI used in most of historical patients was sorafenib, compared to lenvatinib used in this study. Although lenvatinib may have a higher objective response rate and longer progression-free survival than sorafenib (28), the difference between lenvatinib and sorafinib is not large enough to occupy all the difference between the study and historical groups. In this study, DC pulsed by tumor lysate were used to treat post-transplant recurrent HCC, and 9 of 11 patients had stable disease or minor reactions. The disease control rate of DC-immunotherapy is 81.8%, and survival is prolonged in some patients.

DC-based immunotherapy is a safe and promising treatment option for transplant recipients. In this study, patients received 3–10 courses of DC immunotherapy. No adverse events were observed. No DC immunotherapy-related acute rejection was observed. Two patients had minor reactions to DC-immunotherapy, in which the tumors regressed but did not meet the criteria of partial response. One of the two patients underwent surgery because it became feasible to excise the tumors, and the patient has been free from tumors until now. Post-transplant HCC recurrence is an advanced HCC status in patients. Treatment with therapeutic effects and concomitant low adverse effects is important in these patients. In this study, we showed that DC-based immunotherapy has therapeutic effects, with few adverse effects. DC-immunotherapy potentially met the requirements for post-transplant HCC recurrence treatment.

Immune checkpoint inhibitors are used to treat advanced HCC (12, 13, 30). However, whether ICI can be used to treat post-transplant recurrent HCC is concerned because ICI may cause acute rejection and lead to graft loss (15, 16, 31). In this study, three (two prior to and one after DC-immunotherapy) patients received pembrolizumab combined with lenvatinib, but no tumor response was seen in these 3 patients. One patient had a clinical suspicion of acute rejection and required steroid treatment. Clinical data on ICI treatment for post-transplant HCC recurrence are limited. In the literature, ICI used to treat post-transplant HCC recurrence or *de novo* malignancy can yield outcomes similar to those of patients without organ transplantation. However, the incidence of acute rejection was as high as one-third of patients (32, 33). ICI application in transplant recipients should be very careful.

DC immunotherapy is a highly personalized study. The limitations of this DC-immunotherapy highly depend on patient condition which is associated with cell number yielded and response to treatment. The yielded DC number is various in each propagation because monocyte counts are different in different conditions for each patient. Hence, it is believed that DC-therapy administration shall be carried out before immunity is deteriorated. Another limitation of this study is that HCC-specific or associate antigens are difficult to be specified until now and antigen specificity of DC-induced immunity is not performed. The number of patients receiving DC immunotherapy in this study was also limited. Nevertheless, DC immunotherapy is a new treatment for post-transplant HCC recurrence. The short-term outcomes were promising. Long-term follow-up and a larger number of patients are needed in future studies.

In conclusion, HCC recurrence after transplantation is a troublesome issue with poor prognosis. Adding DC-immunotherapy to TKI treatment regimens can significantly prolong survival without causing severe adverse effects. DC immunotherapy can be used to treat post-transplant HCC recurrence.

## Data Availability

The original contributions presented in the study are included in the article/supplementary material. Further inquiries can be directed to the corresponding author.
